# On the phylogeny of Mustelidae subfamilies: analysis of seventeen nuclear non-coding loci and mitochondrial complete genomes

**DOI:** 10.1186/1471-2148-11-92

**Published:** 2011-04-10

**Authors:** Li Yu, Dan Peng, Jiang Liu, Pengtao Luan, Lu Liang, Hang Lee, Muyeong Lee, Oliver A Ryder, Yaping Zhang

**Affiliations:** 1 Laboratory for Conservation and Utilization of Bio-resource & Key Laboratory for Microbial Resources of the Ministry of Education, Yunnan University, Kunming, 650091, PR, China; 2 State Key Laboratory of Genetic Resources and Evolution, Kunming Institute of Zoology, Kunming 650223, China; 3 Conservation Genome Resource Bank for Korean Wildlife, Research Institute for Veterinary Science and Coll. of Vet. Med., Seoul National Univ., Seoul 151-742, South Korea; 4 San Diego Zoo's Institute for Conservation Research, Escondido, CA, USA 92027-7000

## Abstract

**Background:**

Mustelidae, as the largest and most-diverse family of order Carnivora, comprises eight subfamilies. Phylogenetic relationships among these Mustelidae subfamilies remain argumentative subjects in recent years. One of the main reasons is that the mustelids represent a typical example of rapid evolutionary radiation and recent speciation event. Prior investigation has been concentrated on the application of different mitochondrial (mt) sequence and nuclear protein-coding data, herein we employ 17 nuclear non-coding loci (>15 kb), in conjunction with mt complete genome data (>16 kb), to clarify these enigmatic problems.

**Results:**

The combined nuclear intron and mt genome analyses both robustly support that Taxidiinae diverged first, followed by Melinae. Lutrinae and Mustelinae are grouped together in all analyses with strong supports. The position of Helictidinae, however, is enigmatic because the mt genome analysis places it to the clade uniting Lutrinae and Mustelinae, whereas the nuclear intron analysis favores a novel view supporting a closer relationship of Helictidinae to Martinae. This finding emphasizes a need to add more data and include more taxa to resolve this problem. In addition, the molecular dating provides insights into the time scale of the origin and diversification of the Mustelidae subfamilies. Finally, the phylogenetic performances and limits of nuclear introns and mt genes are discussed in the context of Mustelidae phylogeny.

**Conclusion:**

Our study not only brings new perspectives on the previously obscured phylogenetic relationships among Mustelidae subfamilies, but also provides another example demonstrating the effectiveness of nuclear non-coding loci for reconstructing evolutionary histories in a group that has undergone rapid bursts of speciation.

## Background

The Mustelidae is the largest and most-diverse family of Carnivora with a distribution throughout all continents except Australia and Antarctica [1,2]. Recent classifications of the Mustelidae recognize up to eight subfamilies: Mustelinae, Galictinae, Helictidinae, Martinae, Melinae, Lutrinae, Mellivorinae, and Taxidiinae [3-5]. Phylogenetic relationships among these subfamlies have been hotly disputed in pioneer studies [3-15] and are not well established yet. The main problem is that the family Mustelidae represents a typical example of rapid evolutionary radiation and recent speciation event [6,7], dating back to the Oligocene [16-18]. For this reason, attempts to clarify relationships among the eight Mustelidae subfamilies based on a variety of molecular studies have encountered challenges.

**Figure 1:**
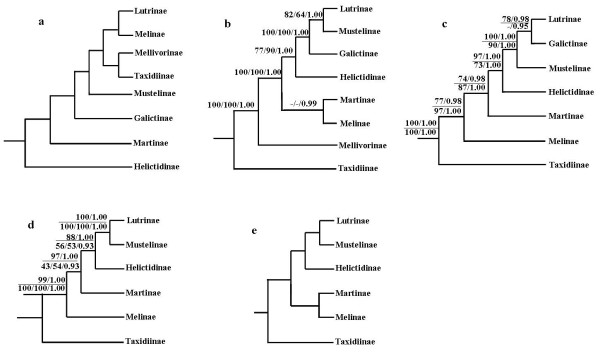
**Hypotheses of phylogenetic relationships among Mustelidae subfamilies**. Trees were reconstructed based on (a) 46 morphological characters [8], (b) analyses of 21 nuclear genes and 1 mt gene [5], (c) analyses of 5 nuclear genes and 1 mt gene [10] (support values are indicated above the line) and 25 nuclear genes and mt genome [13] (support values are indicated below the line), (d) analyses of 5 nuclear genes [4] (support values are indicated above the line) [12] support values are indicated below the line), (e) supertree analyses of 5 nuclear genes [4].

Previously, molecular studies of the phylogenetic reconstruction of subfamilies within the Mustelidae were based on short fragments of nuclear and mt DNA. Recently, some efforts have been made to obtain the phylogenetic tree based on large datasets, including those using 12 mt protein-coding genes [15], 5 nuclear genes [4,12], 4 nuclear genes and 1 mt gene [10,14], 21 nuclear genes and 1 mt gene [5] and 25 nuclear genes and mt genes [13]. Despite numerous efforts, however, evolutionary relationships among the Mustelidae subfamilies remain controversial (see Figure 1).

Hence, it is necessary to exploit larger independent sources of phylogenetic characters to clarify these enigmatic problems. Several studies have shown that relative to the commonly used nuclear protein-coding and mt genes, the noncoding intron sequences can be an equally fruitful source of phylogenetic characters as they possess a number of traits that are desirable for molecular phylogenetics [19-24], for example, lack of functional constraints, a high substitution rate and less homoplasy [19,25,26]. In these studies, the nuclear introns have been shown to provide powerful complementary data to address the ambiguous relationships of different taxonomic levels, including the beaked whale species [23], the Asian pitvipers genus [19], the carnivoran families [24,27], and the eutherian orders [21].

In the present study, we aim to sequence 17 nuclear intron loci comprising a total of >15 kb from 17 mustelids. The mustelids examined here represent all subfamilies of Mustelidae, except for Mellivorinae and Galictinae. Of the 17 nuclear loci, 14 were first applied in the studies of Mustelidae phylogeny. In addition, we undertook the sequencing of the mt genome from these species and presented a phylogeny based on the mt genome data currently available for mustelids. Our objectives were to: (1) provide new insights into the relationships among the Mustelidae subfamilies, and (2) examine the utilities and evolutionary dynamics of the nuclear and mt genes in the context of Mustelidae phylogeny, with special attention to the previously unexplored nuclear intron genes.

## Results

### Characteristics of the Nuclear Intron Data and Mt Genomes

The general characteristics of the nuclear intron data and mt genomes are summarized in Table 1. The 17 nuclear introns of 21 species varied in length from 642 (*Fgb-7*) to 1685 (*Plod2-14*) aligned positions. The removal of ambiguous areas resulted in length variation of the aligned sequences from 523 (Fgb-4) to 1332 (Coro1c-4) positions. The numbers of parsimony-informative sites range from 71 (13.58%) (*Fgb-4*) to 343 (30.25%) (*Cidea-1*). According to different gap selection criteria in Gblocks, the alignment of the combined dataset comprised 15688 (allowed gap positions = all), 15038 (allowed gap positions = with half) and 12570 (allowed gap positions = none) positions. The parsimony-informative sites in these three datasets are 2170 (13.83%), 2137(14.21%) and 1735 (13.80%), respectively. An A-T bias (average = 58.64%) and low transition (Ti)/transversion (Tv) rate ratio (average = 1.93) were observed in most introns. In addition, most introns showed gamma shape parameters (α) close to or larger than 1.0. The nuclear sequence divergence among ingroup taxa ranged from 3% (*Wasf1-7*) to 8.2% (*Tbc1d7-6*), and averaged 4.7%.

**Table 1:** Characterization of Nuclear introns and mt genes examined in the present study

Sequence type	Fragments name	TEs	**Aligned**^**a**^	**Final data**^**b**^	Parsimony-informative sites	Nucleotide Composition				Ti/Tv	Best fit model	Among-site Rate Variation		Pairwise Distance (%)
										
						A	T	G	C			I	α	
Nucear introns	Cidea-1	YES	1590	1134	343	0.308	0.240	0.230	0.222	1.5	TrN+G	0	0.9770	7.5
	Coro1c-4	YES	1435	1332	339	0.242	0.280	0.234	0.243	2.4	K81uf+G	0	0.9379	6.3
	Coro1c-5	YES	1380	1099	140	0.309	0.321	0.210	0.160	2.2	TVM+G	0	0.9084	4.7
	Guca1b-3	YES	711	711	149	0.230	0.186	0.289	0.295	1.6	HKY+G	0	0.7174	6.1
	Ociad1-4	YES	1576	1014	200	0.328	0.393	0.145	0.134	1.9	TVM+G	0	1.3754	4.1
	Plod2-13	NO	1317	1230	236	0.293	0.325	0.229	0.153	2.0	GTR+G	0	1.555	3.3
	Plod2-14	YES	1685	1068	263	0.342	0.327	0.180	0.151	1.4	TVM+G	0	1.4866	3.7
	Ssr1-5	YES	1180	783	131	0.324	0.382	0.160	0.134	2.2	GTR+G	0	1.4043	3.6
	Tbc1d7-6	YES	1153	1095	326	0.247	0.293	0.261	0.199	1.5	TIM+G	0	0.7365	8.2
	Tinagl1-1	YES	1263	1260	250	0.208	0.208	0.295	0.290	2.4	HKY+G	0	0.7637	3.2
	Tinagl1-3	YES	1115	1014	193	0.179	0.238	0.281	0.301	1.9	K81uf+G	0	0.7201	3.7
	Wasf1-3	YES	1195	1021	161	0.314	0.345	0.170	0.171	1.8	GTR+G	0	1.6253	3.9
	Wasf1-6	YES	1276	1077	207	0.308	0.354	0.191	0.147	2.2	TVM+G	0	1.7531	4.1
	Wasf1-7	NO	1380	1147	223	0.314	0.355	0.165	0.166	2.0	GTR+G	0	0.6571	3
	Ttr-1	YES	1130	895	159	0.263	0.279	0.227	0.231	2.4	TVM+G	0	1.3403	4.6
	Fgb-4	YES	847	523	71	0.307	0.307	0.215	0.171	2.0	HKY	0	equal	4.5
	Fgb-7	YES	642	611	102	0.292	0.327	0.186	0.195	1.4	GTR	0	equal	5.1
	
	Com1^c^		15893	12570	1735	0.279	0.301	0.219	0.201	2.0	TVM+I+G	0.17	0.9090	4.4
	Com2^c^		15893	15038	2137	0.279	0.302	0.218	0.201	1.9	GTR+I+G	0.18	0.9078	4.6
	Com3^c^		15893	15688	2170	0.279	0.303	0.218	0.200	1.8	GTR+I+G	0.18	0.9052	4.6

Mt genes	ND1		957		357	0.309	0.274	0.122	0.295	2.6	GTR+I+G	0.53	0.7988	17.3
	ND2		1044		483	0.363	0.263	0.098	0.275	2.1	GTR+I+G	0.35	0.859	23
	COX1		1545		556	0.282	0.302	0.175	0.241	3.1	GTR+I+G	0.58	1.1772	19.1
	COX2		684		252	0.323	0.276	0.142	0.260	3.3	K81uf+I+G	0.56	1.0711	19.6
	ATP8		204		92	0.388	0.291	0.077	0.243	2.2	GTR+I+G	0.42	1.3437	22.8
	ATP6		681		290	0.306	0.291	0.116	0.287	3.3	GTR+I+G	0.46	0.7582	21.6
	COX3		784		303	0.273	0.286	0.154	0.286	2.6	TrN+I+G	0.53	0.8079	20.2
	ND3		348		160	0.320	0.279	0.130	0.272	2.5	TrN+I+G	0.38	0.956	22.3
	ND4L		297		127	0.287	0.324	0.124	0.265	2.9	TVM+I+G	0.43	0.7530	22.5
	ND4		1378		609	0.323	0.278	0.115	0.284	2.8	GTR+I+G	0.42	0.835	22.1
	ND5		1830		739	0.331	0.274	0.115	0.280	2.6	TIM+I+G	0.42	0.8367	19.6
	ND6		534		192	0.417	0.203	0.101	0.278	2.7	K81uf+I+G	0.46	0.656	16.3
	CYTB		1140		446	0.295	0.278	0.134	0.293	2.8	TVM+I+G	0.5	1.1027	18.7
	12SrRNA		988		236	0.371	0.229	0.178	0.223	2.8	GTR+I+G	0.37	0.3532	9.2
	16SrRNA		1626		403	0.369	0.243	0.174	0.213	2.1	GTR+I+G	0.47	0.5765	10.5
	tRNA		1558		301	0.353	0.281	0.157	0.209	4.0	GTR+I+G	0.48	0.4371	7.3
	D-loop		941		315	0.308	0.277	0.166	0.249	1.9	HKY+I+G	0.3	0.4867	11.4
	
	Combined		16537		5855	0.329	0.272	0.140	0.259	2.7	GTR+I+G	0.5	0.9372	16.9

Note: Ti = Transition; Tv = Transversion; I = Proportion of invariable sites; α = Gamma distribution shape parameter; TEs = Transposible Elements. If TEs were detected in introns, it indicated YES, otherwise it indicated NO.

a The length of sequences which were aligned using the CLUSTAL software with default settings.

b The length of analyzed data, after the ambiguous areas of the alignment were removed by Gblocks 0.91b.

c The length of all introns concatenated, after the ambiguous areas of the alignment were removed by Gblocks 0.91b with no gap (Com1), half gap (Com2), and all gap (Com3) parameters.

The complete mt genomes of 25 species ranged from 16388 to 16623 bp in size. Length differences are largely due to the variation in tandem repeats within the control region. All genomes shared the same 13 protein-coding genes, 22 tRNAs genes, 2 rRNAs, and a control region, and also the same gene order. These mt genomes are apparently AT-biased (average = 60.1%). The sequence divergence among ingroup taxa ranged from 16.3 (*ND6*) to 23% (*ND2*) for the protein-coding dataset (average 20.4%), from 9.2 (*12S rRNA*) to 10.5% (*16S rRNA*) for the rRNA dataset (average 9.85%), 7.3% for the *tRNA *dataset, 11.4% for the control region, and 16.6% for the complete dataset.

### Occurrence of Transposable Elements (TEs)

In our intron datasets, pervasive transposable element (TE) insertions were discovered (Table 2), which are mainly non-long-terminal repeat retrotransposons (non-LTR), e.g., long interspersed elements (LINEs), short interspersed elements (SINEs), mammalian-wide interspersed repeats (MIRs), and DNA transposons. MIRs and DNA transposons integrated into the orthologous loci of all examined species, which suggested an ancient origin. The result was consistent with the earlier finding that these two classes of TEs represented remnants or "fossils" of TEs, predating the radiation of mammalian orders, and had long ago become inactive in mammalian lineages [28-30].

**Table 2:** Transposable Elements (TEs) discovered in the present study

Intron Fragments	Species	Transposable Elements (TEs)					
		
		Species-specific			Orthologous		
			
		TEs	Class	Length(bp)	TEs	Class	Length(bp)
Cidea1	Martes penanti	SINEC_b2	SINE/tRNA-Lys	192	L1_Canid_	LINE/L1	54-82
	Mephitis mephitis	SINEC_b1	SINE/tRNA-Lys	188			
	Meles meles	SINEC_b1	SINE/tRNA-Lys	194			
	Arctonyx collaris	SINEC_b2	SINE/tRNA-Lys	194			

Coro1c-4					Tigger12c	DNA/TcMar-Tigger	56-59
					MIRb	SINE/MIR	93-112

Coro1c-5	Lutra lutra	SINEC_b1	SINE/tRNA-Lys	187	SINEC_old	SINE/tRNA-Lys	104-107
					L1ME4a	LINE/L1	65-67

Guca1b-3					MIR	SINE/MIR	58-63

Ociad1-4	Mephitis mephitis	SINEC_b2	SINE/tRNA-Lys	268	MIRc	SINE/MIR	92-122
	Martes flavigula/Martes zibellina/Gulo gulo/Martes foina/Martes amaricana/Martes penanti	SINEC_b1	SINE/tRNA-Lys	175-187			

Plod2-14	Raccoon/Kinkajou	SINEC_b1	SINE/tRNA-Lys	192-195	Kanga1a	DNA/TcMar-Tc2	129-188
					Kanga1c	DNA/TcMar-Tc2	81-92

Ssr1-5	Ailurus fulgens	SINEC_b1	SINE/tRNA-Lys	196			

Tbc1d7-6					MIR	SINE/MIR	134-195

Tinagl1-1	Mephitis mephitis/Ailurus fulgens	MIR3	SINE/MIR	72-76	MIR	SINE/MIR	49-64

Tinagl1-3					MIR	SINE/MIR	85-87

Wasf1-3					L2c	LINE/L2	85-90

Wasf1-6					MER58A	DNA/hAT-Charlie	197

Fgb-4	Taxidea taxus	SINEC_b1	SINE/tRNA-Lys	199			
	Lutra lutra	SINEC_b2	SINE/tRNA-Lys	187			

Fgb-7	Mephitis mephitis	SINEC_b1	SINE/tRNA-Lys	193	MIRb	SINE/MIR	196-198
	Mephitis mephitis	SINEC_b2	SINE/tRNA-Lys	193			

Ttr-1					SINEC_b1	SINE/tRNA-Lys	166-193

The great majority of LINEs and SINEs identified here were members of the L1_Canid (Fc) and CAN SINE groups that have been exclusively found in Carnivora [31-38]. Most of SINEs showed restricted taxonomic distributions and were characterized by sporadic locations in the intronic regions. This suggests that those SINEs emerged after species diversification, likely retaining their ability to retrotranspose.

The insertions of TEs at genomic sites are often considered irreversible and random [39], which suggests that they may be excellent homoplasy-free markers in phylogenetic analyses [40-43]. Here, we identified one SINE insertion shared by *Martes flavigula, Martes zibellina, Martes foina, Martes pennanti, Martes amaricana*, and *Gulo gulo*, supporting their close relationship and the monophyly of Martinae subfamily.

### Occurrence of Intra-individual Allele Heterozygotes (IIAHs)

The overall incidence of intra-individual allele heterozygotes (IIAHs) in our 14 new introns appears universal (Table 3). There were 106 cases of IIAHs in total. Of the 21 species examined, 3 to 11 cases of IIAHs were detected from each intron. IIAHs were observed to be either of equal or variable length. 11 of 14 introns had allele length variant heterozygotes due to a 1-bp indel, with the other nucleotide sites either the same or distinct at 1-11 bp. IIAHs of identical length were discovered in all introns with 1-10 substitutional differences.

**Table 3:** Intra-individual Allele Heterozygotes (IIAHs) detected in the present study

Introns	Species																				
	
	Mephitis mephitis	Ailurus fulgens	Procyon lotor	Potos flavus	Martes flavigula	Martes zibellina	Martes foina	Martes amaricana	Martes penanti	Gulo gulo	Mustela kathiah	Mustela nivalis	Mustela sibirica	Mustela frenata	Mustela putorius	Mustela vison	Arctonyx collaris	Meles meles	Melogale moschata	Taxidea taxus	Lutra lutra
Cidea-1	0/1/0	0/0/0	0/1/0	0/1/0	0/0/0	0/3/0	0/0/0	0/0/0	0/0/0	--	0/0/0	0/0/0	1/1/1	0/0/0	0/0/0	0/0/0	0/3/1	0/2/0	0/1/0	0/0/0	--
Coro1c-4	3/1/0	3/1/0	0/0/0	0/1/0	0/0/0	2/0/1	0/0/0	0/0/0	0/0/0	0/0/0	0/0/0	2/0/0	--	0/0/0	--	0/0/0	0/0/0	4/6/0	0/0/0	0/0/0	2/0/0
Coro1c-5	0/0/0	0/0/0	0/0/0	0/0/0	0/0/0	0/0/0	0/1/0	--	--	--	0/0/0	0/5/0	0/0/0	--	0/5/0	--	0/0/0	0/0/0	--	0/1/0	0/0/0
Guca1b-3	0/0/0	0/0/0	0/0/1	--	5/0/0	0/0/0	0/0/0	0/0/0	4/0/0	0/0/0	0/0/0	3/0/1	0/2/0	0/0/0	2/0/0	0/0/0	4/6/0	0/1/0	0/0/0	1/0/0	0/1/0
Ociad1-4	0/0/0	0/0/0	0/3/0	0/1/1	1/0/1	0/1/0	1/1/0	0/0/0	0/0/0	0/0/0	1/4/0	0/0/0	0/0/0	0/0/0	0/0/0	0/0/0	0/1/0	0/0/0	0/0/0	1/1/0	0/0/0
Plod2-13	0/0/0	1/1/0	0/0/0	4/1/1	0/0/1	2/1/0	0/0/0	0/0/0	0/0/0	0/0/0	1/0/0	0/0/0	0/0/0	1/0/0	1/0/0	0/0/0	0/0/0	1/5/0	3/2/0	0/0/0	0/1/0
Plod2-14	0/1/1	0/0/0	0/0/0	1/6/1	0/3/0	0/1/0	0/1/0	0/0/0	0/0/0	0/0/0	0/0/0	1/1/0	1/0/0	0/0/0	0/0/0	0/0/0	0/0/0	0/0/0	0/0/0	0/0/0	0/0/0
Ssr1-5	0/0/0	0/0/0	0/1/1	0/0/1	0/0/0	0/2/0	0/3/0	0/0/0	0/0/0	0/0/0	0/1/0	0/0/0	0/0/0	0/0/0	1/3/0	0/0/0	0/0/0	0/0/0	0/0/0	0/2/0	0/0/0
Tbc1d7-6	1/2/1	0/0/1	0/0/0	2/1/0	1/0/0	1/2/0	4/2/0	0/0/0	0/0/0	0/0/0	1/0/0	7/4/1	0/0/0	1/0/0	1/0/0	0/0/0	0/0/0	0/0/0	0/0/0	0/1/0	0/0/0
Tinag1-1	0/0/0	0/0/0	0/0/0	3/0/0	2/1/1	3/1/0	0/0/0	0/0/0	1/0/0	0/0/0	3/0/0	2/0/0	0/0/0	0/0/0	3/0/0	0/0/0	0/0/0	0/0/0	3/0/0	0/0/0	1/0/0
Tinagl1-3	0/0/0	0/1/0	0/0/0	1/6/0	1/0/0	0/1/0	0/0/0	0/0/0	0/0/0	0/0/0	0/5/0	0/0/0	0/0/0	0/0/0	0/0/0	0/0/0	1/1/0	1/0/0	1/0/0	0/0/0	--
Wasf1-3	0/0/0	0/0/0	0/0/0	2/0/1	0/0/0	0/2/0	0/0/0	0/0/0	0/0/0	0/0/0	0/0/0	0/3/0	2/0/0	1/0/0	0/0/0	0/0/0	0/0/0	0/0/0	0/0/0	0/0/0	0/0/0
Wasf1-6	0/0/0	0/0/0	3/1/0	2/0/0	0/0/0	0/0/0	0/0/0	--	0/0/0	0/0/0	0/0/0	0/0/0	0/0/0	0/0/0	0/0/0	0/0/0	0/0/0	0/0/0	0/0/0	1/0/0	0/0/0
Wasf1-7	0/0/1	1/0/1	2/0/0	1/1/0	1/0/0	0/0/0	1/0/0	0/0/0	0/0/0	0/0/0	0/0/0	7/1/0	0/0/0	0/0/0	0/0/0	0/0/0	0/0/0	0/0/0	3/0/0	1/0/0	0/0/0

Notes: The information of IIAHs were represented as transitions/transversions/indels. The numbers correspond to the number of transitions, the number of transversions and the length of indels, respectively. -- stands for unknown numbers due to the unavailiablity of sequences data

**Figure 2:**
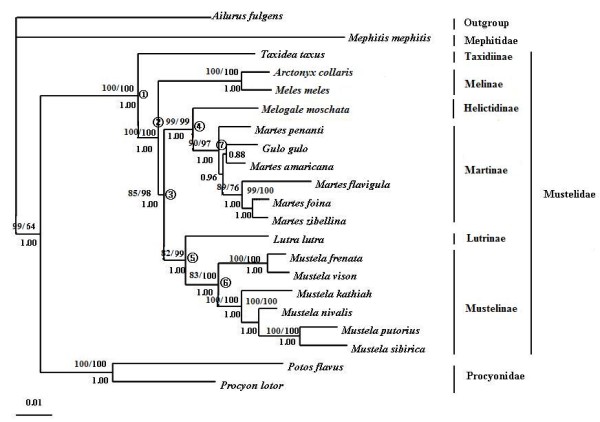
**MP/Bayesian/ML phylogenetic tree of 21 mustelidae species for 17 combined nuclear intron loci**. MP/ML bootstrap values (allowed gap positions = all in Gblocks) are shown above nodes. Posterior probabilities are shown below nodes. Node numbers that were used in the divergence time estimations and phylogenetic performance evaluation are indicated in the tree.

Generally, IIAHs formed monophyletic pairs on the phylogenetic trees as expected (see Additional file 1). Two cases of strongly-supported nonmonophyletic IIAHs were illustrated by the close relatedness of one allele of the least weasel *Mustela nivalis *to one allele of the European polecat *Mustela putorius *(*Plod2-13 *and *Guca1b-3 *genes; Figure 2), which are most likely to indicate the cases of incomplete lineage sorting.

### Phylogenetic Inference

Although individual nuclear gene analyses produced inconsistent topologies with low levels of support (Additional file 1), possibly due to limited phylogenetic information harbored in a single gene, the analyses of the combined nuclear data set using three gap selection criteria in Gblocks (allowed gap positions = none, with half, and all) and different tree-building methods (MP, ML and Bayesian methods) yielded nearly identical, well-resolved trees with strong support for all nodes, except for the relationships among *Gulo gulo, Martes americana*, and *Martes pennanti *(Figure 2). In the tree, Taxidiinae diverged first (MP BS = 100%, ML BS = 100%, PP = 1.00), followed by Melinae (MP BS = 85%, ML BS = 98%, PP = 1.00). The remaining mustelids were divided into two clades, one consisting of Martinae and Helictidinae (MP BS = 99%, ML BS = 99%, PP = 1.00), and the other one consisting of Lutrinae and Mustelinae (MP BS = 82%, ML BS = 99%, PP = 1.00). In addition, both the inclusion of IIAHs in the combined nuclear analysis using software POFAD [44] and the Bayesian concordance analysis (BCA) analysis (Additional file 2) using software BUCKy [45] produced the same tree topologies as that in Figure 2. At the subfamily level, all nodes in the BCA analysis received high concordance factors (CF) value (1.000).

**Figure 3:**
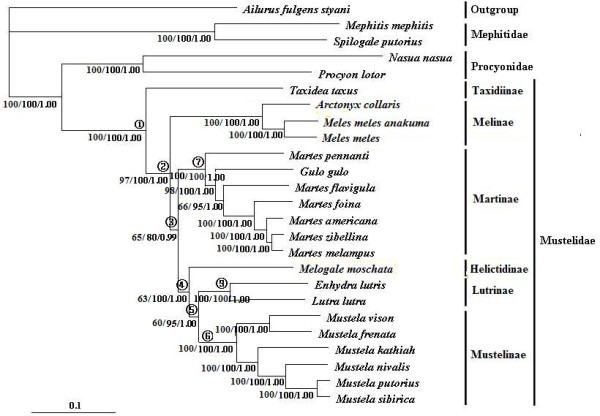
**MP/Bayesian/ML phylogenetic of 25 mustelidae species for the complete mt genomes**. MP/ML bootstrap values/Posterior probabilities are shown below nodes. Node numbers that were used in the divergence time estimations and phylogenetic performance evaluation are indicated in the tree.

For individual mt gene analyses, the rRNA and tRNA data sets demonstrated reduced resolving power for phylogenetic inference compared to protein-coding gene analysis (Additional file 3). The complete mtDNA genome-based analyses, irrespective of the used tree-building methods and parameter sets, produced a well-resolved and well-supported tree (Figure 3), with the tree topology and branch supports identical to that of the combined protein-coding gene analysis. At the subfamilial level, the single difference between mt genome tree (Figure 3) and combined nuclear gene tree (Figure 2) is the phylogenetic position of Helictidinae. In mt genome tree, Helictidinae is closer to the clade uniting Lutrinae and Mustelinae than to Martinae (MP = 60%; ML BS = 95%; PP = 1.00).

The phylogenetic tree reconstructed from the combined nuclear and mt genome data set by using the BCA analysis [45] produced a tree topology (Additional file 2) identical to that from the combined nuclear gene analysis (Figure 2). At the subfamily level, all nodes received high CFs (1.000), except for that of Helictidinae (CF = 0.502), whose position is the single discrepancy between the combined nuclear and mt gene trees.

Shimodaira-Hasegawa (SH) test and the approximately unbiased (AU) test were carried out to examine the degree of significant difference between the nuclear and mt trees produced in the present study. Both tests indicated significant topological incongruence concerning the phylogenetic position of Helictidinae between the nuclear and mt trees. When using nuclear data, the mt genome tree topology, in which Helictidinae is closer to the clade uniting Lutrinae and Mustelinae, was rejected by the AU and SH tests (*P *< 0.05). When using mt genome data, the nuclear tree topology, in which Helictidinae and Martinae are grouped together, was rejected by the AU and SH tests (*P *< 0.05).

### Divergence Time Estimation

Divergence time estimates for the origin and diversification of Mustelidae subfamilies yielded broadly consistent results between combined nuclear and mt genome data set (Table 4). Combined nuclear gene analysis placed the earliest branching Taxidiinae around 23.73 Mya (95% confidence intervals = 22.80-24.70 Mya). After that, Melinae diverged from the other mustelids at 17.33 Mya (95% confidence intervals = 12.44-22.10 Mya). The split between Martinae/Helictidinae and Lutrinae/Mustelinae took place at 16.05 Mya (95% confidence intervals = 11.34-20.91 Mya). The divergence between Martinae and Helictidinae occurred at 10.21 Mya (95% confidence intervals = 5.66-15.04 Mya), and that between Lutrinae and Mustelinae at 11.43 Mya (95% confidence intervals = 7.02-16.00 Mya). The origins of Mustelinae and Martinae were estimated to be 5.89 Mya (95% confidence intervals = 4.99-6.83 Mya) and 5.97 Mya (95% confidence intervals = 3.39-8.90 Mya), respectively.

**Table 4:** Divergence time estimations (in millions of years ago) for major diversification events of Mustelidae, including data from previous studies

Node	Diversification event	**Nuclear data**^**a**^**(95% CI)**^**c**^	**Mt data**^**b**^**(95% CI)**	Fossil record	Wayne (1989)	Bininda-Emonds (1999)	Hosoda (2000)	Sato (2003)	Yonezawa (2007)	Koepli (2008)	Eizirik et al (2010)
1	divergence of Taxidiinae to the other mustelids	23.73(22.80-24.70)	23.46(22.50-24.41)	-	-	20.8	-	-	20.21(18.78-21.64)	24.2(22.3-26)	13.0 (9.6-17.1)
2	divergence of Melinae	17.33(12.44-22.10)	17.19(13.96-20.58)	18-20.5	-	13.7	-	14.5-18.1	16.98(15.52-18.44)	11(9.4-12.5)	11.7 (8.5-15.5)
3	origin of the other mustlids except for Taxidiinae and Melinae	16.05(11.34-20.91)	15.79(12.65-19.11)	21.5-22.5	-	11.4	-	14.7-14.8	14.83(13.39-16.27)	11.6(10.1-13)	10.8 (7.8-14.5)
4	divergence of Helictidinae	10.21(5.66-15.04)	13.86(10.45-17.02)	-	-	6.9	-	-	-	10.8(9.4-12.2)	-
5	divergence between Lutrinae and Mustelinae	11.43(7.02-16.00)	12.54(9.24-15.87)	11.1-13.5	20-25	9.9-17.1	15-23	13.5-14.1	12.74(11.41-14.07)	8.7(7.3-10.0)	8.4(5.9-11.5)
7	origin of Mustelinae	5.89(4.99-6.83)	6.30(5.39-7.24)	3.4-4.2	12-20	10.4-11.4	10-23	8.5-9.9	-	6.1(4.9-7.2)	-
6	origin of Martinae	5.97(3.39-8.90)	10.91(7.82-14.37)	3.3-4.0	-	8.2	10-14	-	11.48(10.15-12.81)	6.8(5.1-8.5)	7.7(5.3-10.8)

Node numbers correspond to those indicated in Figure 2 and 3

a the divergence times were estimated based on combined nuclear genes and tree topology in Figure 2

b the divergence times were estimated based on mt genomes and tree topology in Figure 3

c 95% confidence intervals

The analyses of the mt genome data set suggested the origin of Taxidiinae around 23.46 Mya (95% confidence intervals = 22.50-24.41 Mya). Other time estimates included the split of the Melinae clade from other mustelids at 17.19 Mya (95% confidence intervals = 13.96-20.58 Mya), the separation between Helictidinae/Lutrinae/Mustelinae and Martinae about 15.79 Mya (95% confidence intervals = 12.65-19.11 Mya), the divergence between Helictidinae and Lutrinae/Mustelinae at 13.86 Mya (95% confidence intervals = 10.45-17.02 Mya), and the divergence between Lutrinae and Mustelinae at 12.54 Mya (95% confidence intervals = 9.24-15.87 Mya). The origins of Mustelinae and Martinae were estimated to be 6.30 Mya (95% confidence intervals = 5.39-7.24 Mya) and 10.91 Mya (95% confidence intervals = 7.82-14.39 Mya), respectively.

## Discussion

### Phylogeny of Mustelidae Subfamilies

Among mammalian phylogenies, those characterized by rapid species radiations have long been one of the plaguing and challenging problems in species tree reconstruction [46]. This is the first study utilizing data from such large-scale nuclear non-coding loci from Mustelidae.

Both our combined nuclear intron and mt genome phylogenies not only strongly favor the prevailing view that Taxidiinae was the most basal member within family Mustelidae [4,5,10-14], but also provide strong evidence that Melinae diverged between Taxidiinae and all the other mustelids examined as well. The latter is in contradiction to morphological investigations [8], but supports the nuclear gene results from Sato et al. [11,12] and Wolsan and Sato [13], and disagree those from Koepfli et al. [5] and Yu et al. [15].

Notably, the sister relationship between Lutrinae and Mustelinae was reinforced by consistent recovery from both our mt genome and combined nuclear intron analyses with high confidence, upholding and strengthening the hypothesis drawn by almost all sequence-based analyses in previous studies [4,5,7,10-15]. In contrast, the position of Helictidinae varied between our nuclear and mt genome analyses. Nuclear data analysis placed it as sister to Martinae, whereas mt genome data indicated a sister-taxa association of it to the clade uniting Lutrinae and Mustelinae. Our mt genome result (Figure 3) is consistent with that inferred from most previous nuclear studies [3-5,10-13], but dissented from morphological view and karyological analyses [8,47-49]. Interestingly, our combined nuclear analysis (Figure 2) yields a result that is different from all previous hypotheses, suggesting for the first time Helictidinae and Martinae are more closely related to each other than any other taxa in Mustelidae.

Corresponding tests (AU and KH tests) have indicated significant topological incongruence between nuclear and mt trees. When using nuclear data, the mt genome tree topology was rejected by the AU and SH tests (*P *< 0.05), and vice versa. Phylogenetic incongruence between nuclear and mitochondrial genes has also been reported in *Drosophila*, Aves and bears [50-53]. Various elements may bear the responsibility for the presence of conflicting signal regarding the placement of Helictidinae, including different evolutionary histories and gene properties in gene regions from different genomes, sampling error and lineage sorting. The probability of their occurrence increased especially when separation time between different species is short [54-57], as in the present study. Although the BCA analysis of the combined nuclear intron and mt genome sequences, which is an approach that allows for gene tree discordance, retrieved an identical tree topology to that of the nuclear intron gene, the position of Helictidinae received week support. Therefore, the analyses involving more characters in the future may help to confirm the precise position of Helictidinae.

### Implications for Mustelidae Radiation

Mustelidae has one of the most extensive fossil records of extant Carnivora families [8], and molecular dating of the Mustelidae radiation has also been attempted in several studies previously. Our results, from an independent character source, provide important insights into the time scale of the origin and diversification of extant subfamilies of Mustelidae. It is interesting to draw a comparison of the dating results estimated from prior and present studies (Table 4).

Among the subfamilies, Taxidiinae is the basal-most branching lineage in mustelid diversification dating to the late Oligocene, which is more concordant with the time estimate of Koepfli et al. [5], but earlier than those of Bininda-Emonds et al. [58], Yonezawa et al. [15] and Eizirik et al. [59]. The divergence time of the next branching lineage, i.e., Melinae clade, is dated to the early Miocene in our analysis, which is more broadly consistent with the paleontological data [60] and the sequence-based data from Yonezawa et al. [15] than those from Sato et al. [2], Bininda-Emonds et al. [54], Koepfli et al. [5], and Eizirik et al [59]. The estimates of the origin of the remaining mustelids are more recent than those fossil-based ages [61], and older than the other sequence-based dates [2,5,15,58,59].

Among the remaining mustelids, one important event of the mustelid diversification is the divergence between Lutrinae and Mustelinae. Our estimated dates are consistent with the corresponding fossil records [60,62] and that from Yonezawa et al. [15], but more recent than those from Hosoda et al. [63], Wayne [64], and Sato et al. [2], and older than those from Koepfli et al. [5] and Eizirik et al. [59]. Other important events are the origins of Mustelinae and Martinae. Our estimates for them are both much older than the existing fossil record [65,66]. The dates of Mustelinae origin are older than most of the other molecular estimates, but in good agreement with that of Koepfli et al. [5]. As regards the origin of Martinae, there is large difference between our nuclear and mt genome estimates. The nuclear estimate is more in agreement with those from Koepfli et al. [5] and Eizirik et al. [59], while the mt estimate is more concordant with those from Hosoda et al. [63] and Yonezawa et al. [15].

In addition, our analyses resulted in time estimates of divergence of Helictidinae that more agree with that from Koepfli et al. [5] than that from Bininda-Emonds et al. [58], which is younger than the present results. More intensive taxonomic sampling will improve accuracy on the time estimation of mustelid diversification.

### Utilities of the nuclear introns in phylogenetic study of Mustelidae subfamilies

Several recent studies have indicated that nuclear introns hold considerable signals for resolution of difficult phylogenies at both shallow and deeper species level hierarchies [21-24,27,67,68]. We are among the first to use large-scale nuclear intron genes in inferring phylogenies of Mustelidae. Our analysis not only brings new perspectives on the phylogenetic relationship of Mustelidae subfamilies, but provides another example demonstrating that the nuclear non-coding genes can be an effective data source for reconstructing evolutionary histories in a group that has undergone rapid bursts of speciation as well.

We assessed the phylogenetic utilities of individual introns and mt genes in resolution of the inter-subfamilial relationships of Mustelidae by counting the number of congruent nodes between the individual phylogenies and the combined gene trees (Table 5). In the individual nuclear gene analyses, the *Plod2-13 *gene recovered all 8 nodes of the combined nuclear gene tree. Anyway, the *Plod2-13 *and *Plod2-14 *genes recovered the highest number of congruent nodes of the combined nuclear gene tree, whereas the *Wasf1-7, Guca1b-3, Ssr1-5*, and *Tbc1d7-6 *genes showed the lowest phylogenetic performance. As regards the mt gene analyses, we observed that the *ND5 *and *CYTB *genes recovered all 9 nodes of the mt genome tree. Ranking the single mt gene shows that the *ND5, CYTB, 16SrRNA *and *ND2 *genes are better indicators of Mustelidae phylogeny at subfamilial level than are other genes, such as *Atp8, Atp6 *and *ND4L *genes. This result agrees broadly with previous conclusions about the rough classification of mt genes into good, medium, and poor performance categories [69-74] (Additional file 4). In summary, the assessment of phylogenetic utility and limits of these individual nuclear and mt genes makes it possible to preselect subsets of genes for future molecular studies of vertebrate phylogeny.

**Table 5:** Phylogenetic performance of nuclear and mt genes

**Gene**^**a**^		no. congruent branches (BP > 0.95)	no. congruent branches (BP < 0.95)	total no. congruent branches	**Node**^**b**^								
					
					1	2	3	4	5	6	7	8	9
Mt gene	nd5	8	1	9	*	*	*	**#**	*	*	*	*	*
	cytb	8	1	9	*	*	*	*	**#**	*	*	*	*
	16sRNA	8	0	8	*	*		*	*	*	*	*	*
	nd2	6	1	7	*		#	*		*	*	*	*
	cox2	6	0	6	*		*	*		*	*	*	
	nd4	6	0	6	*				*	*	*	*	*
	nd6	5	1	6	*	*				*	*	*	**#**
	12sRNA	5	1	6	*	#				*	*	*	*
	nd3	4	2	6	*	#				*	**#**	*	*
	cox1	5	0	5	*					*	*	*	*
	cox3	5	0	5	*	*		**#**		*	*	*	
	tRNAs	4	1	5	*	#					*	*	*
	nd1	4	1	5	*					**#**	*	*	*
	atp8	3	1	4	*	#				*		*	
	atp6	4	0	4	*					*		*	*
	nd4l	2	0	2						*	**#**	*	

Nuclear gene	Plod2-13	6	2	8	*	*	#	**#**	*	*	*	*	
	Plod2-14	6	1	7	*	*	*	*	**#**	*		*	
	Coro1c-5	5	1	6	*	*		*	**#**	*	**-**	*	
	Tinagl1-3	5	1	6	*	*	#	*	**-**	*		*	
	Coro1c-4	6	0	6	*		*		*	*	*	*	
	Tinagl1-1	6	0	6	*	*		*	*	*		*	
	Cidea-1	6	0	6	*	*	*	*	**-**	*		*	
	Ociad1-4	4	2	6	*	#	*	*	**#**			*	
	Wasf1-3	5	1	6	*	*		*	**#**	*		*	
	Fgb-7	5	1	6	#	*		*	*	*		*	
	Ttr-1	6	0	6	*	*		*	*	*	**-**	*	
	Wasf1-6	5	0	5	*	*		*	*			*	
	Fgb-4	4	1	5	*			*	**#**	*		*	
	Wasf1-7	3	0	3	*	*						*	
	Guca1b-3	2	0	2	*							*	
	Ssr1-5	2	0	2	*							*	
	Tbc1d7-6	0	0	0									

Node numbers correspond to those indicated in Figure 2 and 3

a genes are ranked by the total number of congruent branches in the combined topologies

b there are 9 nodes in total indicated in the mt genome tree (Figure 3) and 8 nodes in the combined nuclear gene tree (Figure 2)

* branches with PP > 0.95 congruent in the combined topology

# branches with PP < 0.95 congruent in the combined topology

Although the use of nuclear introns as genetic markers has now been implemented as a powerful approach in recent phylogenetic studies, there are, however, numerous potential problems associated with this approach. Chief among these is the difficulties in sequence data acquisition, alignment, and analysis of the nuclear introns compared to the traditional mt and nuclear protein-coding genes, as a result of the higher rates of variation and frequencies of indels. The common presence of indels (including TEs, small gaps and tandem repeats) and IIAHs, as reported in this study, makes experimental work labor-intensive by virtue of the additional time and money required to isolate alleles and optimize PCR amplification and sequencing. In addition, they can create positional homology problems associated with areas of ambiguous alignment [75]. Several studies have also shown that the phylogenetic inference is sensitive to the various treatments of indels. These issues are central to the appropriate application of intron data in phylogenetic reconstruction and they should be comprehensively and explicitly addressed in the future studies [19].

## Conclusions

The phylogenetic relationships among Mustelidae subfamilies have posed one of the major problems concerning Carnivora systematics. In this study, phylogenetic relationships among Mustelidae subfamilies are presented based on 17 nuclear intron loci and mt whole genomes. Our results resolve some of the ambiguous issues in Mustelidae phylogeny, whereas some phylogenetic relationships require confirmation by analyzing additional samples and character information, such as the precise position of Helictidinae. Our study not only brings new perspectives on the previously obscured phylogenetic relationships among Mustelidae subfamilies, but also provides another example demonstrating the effectiveness of nuclear non-coding loci for reconstructing evolutionary histories in a group that has undergone rapid bursts of speciation.

## Methods

### Sequence Data

Detailed information of the 14 nuclear intron loci first used in the Mustelidae phylogeny is shown in Additional file 5. These loci were amplified with primers as described in Yu et al. [27] from 17 mustelids and 4 non-mustelid carnivoran species. Three other nuclear introns (*Ttr-1, Fgb-4 *and *Fgb-7*), which were available from our previous published studies [14,76,77], were also included in the present nuclear data set.

The species examined in this study and their Genbank accession numbers are listed in Additional file 6. A "touch-down" PCR amplification was carried out using the following parameters: 95°C hot start (5 min), 10 cycles of 94°C denaturation (1 min), 60-50°C annealing (1 min), 72°C extension (1 min), and finally 25 cycles of 94°C denaturation (1 min), 50°C annealing (1 min), 72°C extension (1 min). The amplified DNA fragments were purified and sequenced in both directions with an ABI PRISM(tm) 3730 DNA sequencer following the manufacturer's protocol. In the case of poor performance of direct sequencing resulting from complex DNA structures, tandem repeats or intron heterozygotes, the amplified PCR products were gel-purified and cloned into the pMD18-T vector (TaKaRa Biotechnology Co., Ltd. Dalian, China) and transformed into ultracompetent *E. coli *cells (TaKaRa Biotechnology Co., Ltd. Dalian, China). Thirty positive clones per ligation reaction were sequenced. All sequences obtained were checked carefully and queried in BLAST searches of GenBank to assess homology. In a few cases, PCR attempts using different primer pairs and cloning methods failed to produce sequence data (see Additional file 6). These sequences were excluded from the independent gene analyses and treated as missing data in the combined analyses. The newly determined nuclear sequences have been deposited in GenBank with accession numbers HM063147-HM063412.

The mt complete genome sequences were amplified using LA PCR™ Kit (Takara Biotechnology Co., Ltd) and 9 universal long PCR primers from 14 mustelids and 2 non-mustelid carnivoran species (Additional file 6). In addition, 23 species-specific primers were designed when the universal PCR primers failed to produce successful PCR amplification. A "touch-down" long PCR amplification was carried out using the following parameters: 95°C hot start (2 min), 10 cycles of 98°C denaturation (10 sec), 67-58°C annealing (1 min 30 sec; °C/cycle), 72°C extension (5 min), and finally 25 cycles of 98°C denaturation (10 sec), 58°C annealing (1 min 30 sec), 72°C extension (5 min). At the end, a final 10-min extension at 72°C was performed. Long PCR products were sequenced in both directions using a primer walking strategy with a total of 306 primers. Sequencing was performed in an ABI PRISM(tm) 3700 DNA sequencer following the manufacturer's protocol. Primer sequence information is available upon request. Where necessary, PCR products were cloned into the pMD18-T vector and transformed into ultracompetent *E. coli *cells (TaKaRa Biotechnology Co., Ltd. Dalian, China) in order to resolve the difficulty of direct sequencing of control regions arising from long tandem repeats. Five positive clones per ligation reaction were sequenced. Mt sequences obtained were checked to ensure that they did not include nuclear copies of mtDNA-like pseudogenes. In addition to these new 16 mt genomes, 6 other mustelid and 3 other non-mustelid mt genomes available in public database were included in the mt analyses (Additional file 6).

### Alignments and sequence Characterizations

Sequences were aligned using CLUSTAL X under the default settings [78]. The nuclear alignment was divided into two data sets: (1) each of the 17 intron loci and (2) combined sequences of all introns. The mt alignment was divided into four data sets: (1) each of the 13 protein-coding genes, (2) 22 tRNAs, (3) two rRNAs, and (4) combined sequences of the tRNAs, rRNAs and protein-coding genes. Due to the presence of ambiguous areas in the nuclear alignments, we excluded the alignment ambiguities using Gblocks 0.91b [79] with default parameters (allowed gap positions = all).

Pairwise comparisons and sequence characterizations were estimated using MEGA 4.0 [80]. Given that nuclear introns tend to be favorable chromosomal regions for integration of transposable elements (TEs) [81], they were screened for interspersed repeats by using the program RepeatMasker (Smit, Hubley and Green, RepeatMasker Open-3.0. 1996-2004, http://www.repeatmasker.org).

### Phylogenetic Analyses

Phylogenetic analyses of the individual introns and mt genes, i.e., nuclear data set (1) and mt data set (1), (2), and (3), were performed using PAUP* 4.0b10 [82] for maximum parsimony (MP) and maximum likelihood (ML) analyses, and using MrBayes 3.1.2 [83] for the Bayesian inference. In MP analyses, a heuristic search was performed with tree-bisection-reconnection (TBR) branch swapping, random addition of taxa, and 1000 replicates per search. Only one of the best trees found during branch swapping was saved. In ML analysis, the best-fit models of sequence evolution were selected using the Akaike Information Criterion (AIC) [84,85] with Modeltest version 3.7 [86]. The chosen models and their parameters were used to infer ML trees with the heuristic algorithm, 10 random-addition sequence replicates, and TBR branch swapping. The tree reliability under ML analysis was assessed using a bootstrap resampling of 100 replicates (BP) [87]. In Bayesian inference, each Metropolis-coupled Markov chain Monte Carlo (MCMC) run for all individual genes employed the model selected by ModelTest for that gene, or the nearest model to that model that could be implemented in MrBayes. Three heated chains and a single cold chain were used in all MCMC analyses and run for 2 × 10^6 ^generations, Trees were sampled every 100 generation. The average standard deviation of split frequencies was close to 0.001 when the run was end. The first 25% were discarded as the burn-in. A 50% majority-rule consensus of post burn-in trees was constructed to summarize posterior probabilities (PP) for each branch.

In addition to individual analyses, phylogenetic reconstruction were performed based on the combined data sets, i.e., nuclear data set (2) and mt data set (4), using PAUP* 4.0b10 [82] for MP analysis, RAxML online web server [88] for partitioned ML analysis and using MrBayes [83] for partitioned Bayesian analysis (pBI) [89]. For the combined data sets, we identified model partitions based on partitioning matrices by locus. That is, in the analysis of combined nuclear data set, each nuclear intron gene was considered as a different partition, whereas in that of combined mt data set, each of the 13 individual protein-coding genes, all tRNAs, and each of the two rRNA genes were considered as different partitions. Based on the selected models using the AIC [84] as mentioned above for individual analyses, we assigned a separate substitution model for each of the data partitions. Three heated chains and a single cold chain were used in all MCMC analyses and run for 5 × 10^6 ^generations, sampling trees every 100 generations. The average standard deviation of split frequencies was close to 0.001 when the run was end. The first 25% were discarded as the burn-in. A 50% majority-rule consensus of post burn-in trees was constructed to summarize posterior probabilities (PP) for each branch.

In addition, given the heterogeneous gene trees observed among 17 nuclear intron gene analyses and between the combined nuclear intron and mt genome analyses, Bayesian concordance analysis (BCA) [45] implemented in the program BUCKy [45], which uses individually calculated gene trees to infer the species tree that maximizes the bipartition concordance among each gene tree, was also performed for both the nuclear intron gene datasets and the nuclear plus the mt genome datasets. Tree reliability was evaluated by sample concordance factors (CFs). The MCMCMC sampled with 2 × 10^6 ^generations was employed (4 runs and 4 chains) and a priori level of discordance α = 2.5 was used in BCA.

In all analyses, trees were rooted with the red panda *Ailurus fulgens *and the skunk *Mephitis mephitis*, based on the general consensus that they branched off earlier than the mustelids [2,4,11,12,76,77].

### Intra-individual allele heterozygotes

For individual intron analyses, both copies of alleles from a species were included. For combined nuclear data sets, we performed phylogenetic analyses by (1) choosing randomly an allele per species for portioned ML analysis and Bayesian analysis (without the inclusion of IIAHs), and (2) using POFAD v1.03 algorithm (Phylogeny Analysis From Allelic Data) [90] to incorporate IIAHs. POFAD is a recently developed method of constructing phylogeny from multiple datasets that contain allelic information. It converts a distance matrix of alleles into a distance matrix of organisms so that individuals become the terminals of the analyses. First, we calculated the average uncorrected pairwise distances in PAUP* [82]. These distances then served as the input for the calculation of standardized pairwise distances between species in POFAD [90]. The standardized distances were then used as input for the neighbor-joining analysis conducted using PAUP* [82] to produce a phylogenetic tree.

### Divergence time estimation

Divergence times based on combined nuclear intron and combined mt data sets were estimated using the Bayesian relaxed phylogenetic approach implemented in BEAST v1.5.4 [91]. We assumed a GTR+I+G model of DNA substitution with four rate categories. Uniform priors were employed for GTR substitution parameters (0, 100), gamma shape parameter (0, 100) and proportion of invariant sites parameter (0, 1). The uncorrelated lognormal relaxed molecular clock model was used to estimate substitution rates for all nodes in the tree, with uniform priors on the mean (0, 100) and standard deviation (0, 10) of this clock model. We employed the Yule process of speciation as the tree prior and a UPGMA tree to construct a starting tree, with the ingroup assumed to be monophyletic with respect to the outgroup.

Three calibration points from the fossil records were applied in the dating analyses. These calibration points are all implemented as minimum age constraints, including 27.6 Mya for the split between Procyonidae and Mustelidae [12,15,92], 24 Mya for the crown Mustelidae [2,5,17], and 5.3 Mya for the origin of the genus Mustela in Mustelinae [5,93]. All fossil constraint priors were set as means of a normal distribution, with a standard deviation of 1.0 MYA. Two independent MCMC runs of 30,000,000 generations were performed for each data set with parameters logged every 1,000 generations. The Auto Optimize Operators function was enabled to maximize efficiency of MCMC runs. Two independent MCMC runs for each analysis were combined to estimate the posterior distribution of the substitution model and tree model parameters, as well as node ages. Analyses of these parameters in Tracer 1.5 [94] suggested that the number of MCMC steps was more than adequate, with effective sample sizes of all parameters often exceeding 1,000 and Tracer plots showing strong equilibrium after discarding burn-in.

### Testing Tree Incongruence

The incongruence among different tree topologies was evaluated using the Shimodaira-Hasegawa (SH) test [95] and the approximately unbiased (AU) test [96], as implemented in the CONSELV0.1i program [97] with default scaling and replicate values. The site-wise log-likelihood values were estimated by PAUP* [82].

## Authors' contributions

LY, YPZ and OAR designed the study and wrote the manuscript. DP and JL carried out the experiment work and performed the sequence analyses. PTL and LL contributed to the experiment work and sequence analyses. HL and ML helped collect samples. All authors read and approved the final manuscript.

## Supplementary Material

Additional file 1 **Phylogenetic relationships of Mustelidae based on the analyses of 17 single introns**. The IIAHs within a species was shown as 1 and 2. All trees shown were reconstructed using Bayesian method. Posterior probabilities (PP) are shown above internal nodes. Click here for file

Additional file 2 **Phylogenetic relationships of Mustelidae based on the Bayesian concordance analysis (BCA) of the nuclear intron gene datasets and the nuclear plus the mt genome datasets**. The concordance factors (CFs) from the nuclear intron gene analysis and the nuclear plus the mt genome analysis are shown above internal nodes. Click here for file

Additional file 3 **Phylogenetic relationships of Mustelidae based on the analyses of 13 individual protein-coding genes, 2 individual rRNA genes, 22 tRNAs, combined protein-coding genes, combined rRNA genes, and combined tRNA genes**. All trees shown were reconstructed using Bayesian method. Posterior probabilities (PP) are shown above internal nodes. Click here for file

Additional file 4 **Comparisons of phylogenetic performances of mt genes among studies**. Click here for file

Additional file 5 **Detailed information of 14 nuclear intron loci that were first used in the Mustelidae phylogeny**. Click here for file

Additional file 6 **The species examined in this study and their Genbank accession numbers**. Click here for file
