# Supplementary material for: On the phylogeny of Mustelidae subfamilies: analysis of seventeen nuclear non-coding loci and mitochondrial complete genomes
Source: BMC Evol Biol. 2011 Apr 10;11:92. doi: 10.1186/1471-2148-11-92 (PMC3088541; doi:10.1186/1471-2148-11-92)

**Additional file 1**

Phylogenetic relationships of Mustelidae based on the analyses of 17 single introns. The IIAHs within a species was shown as 1 and 2. All trees shown were reconstructed using Bayesian method. Posterior probabilities (PP) are shown below internal nodes.

**Coro1c-4**

**Coro1c-5**


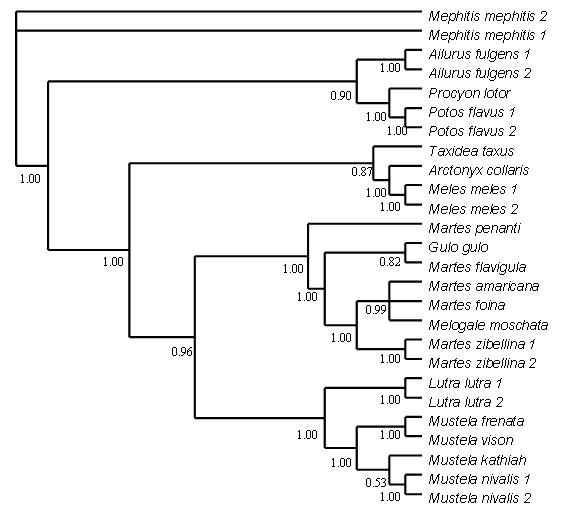

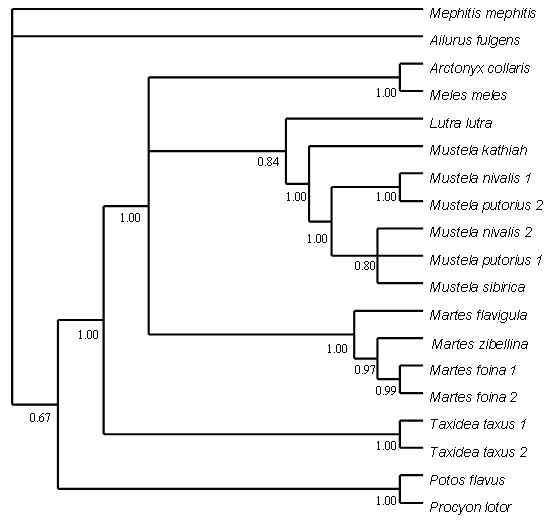


**Ociad1-4**

**Fgb4**


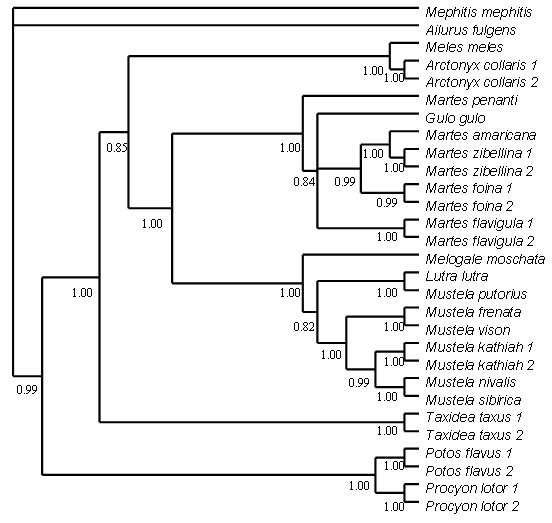

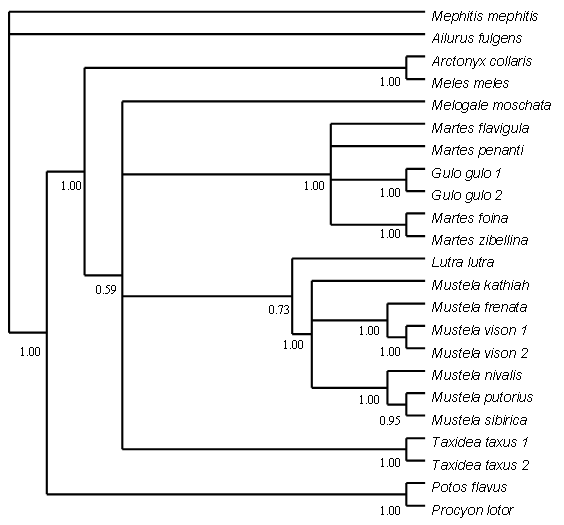


**Fgb7**

**Guca1b-3**


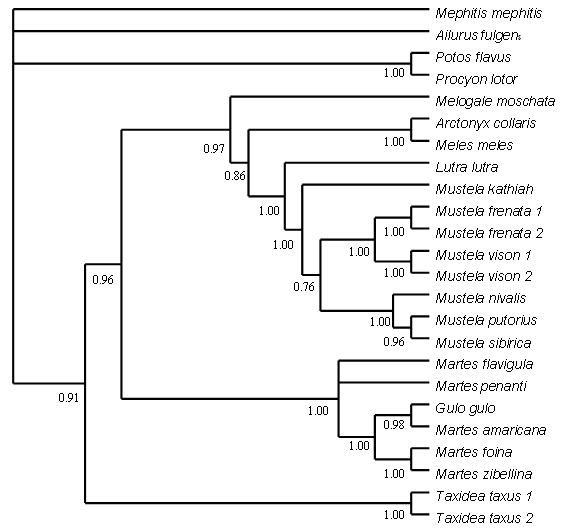

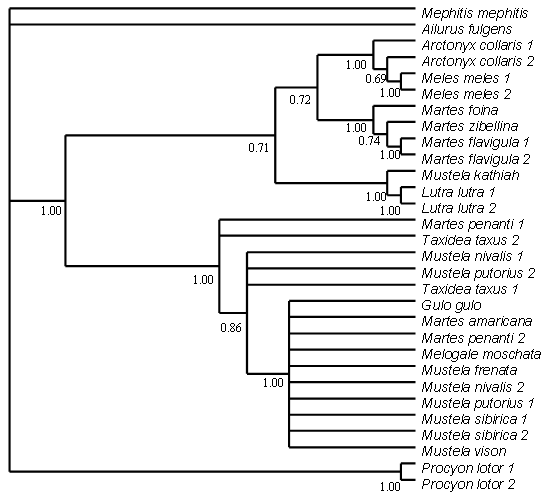


**Cidea1**

**Plod2-13**


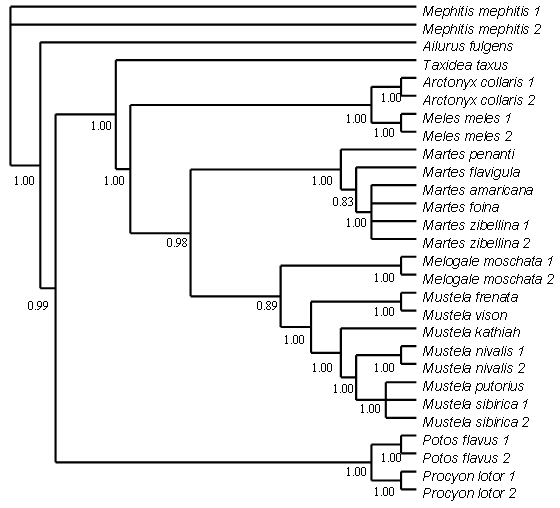

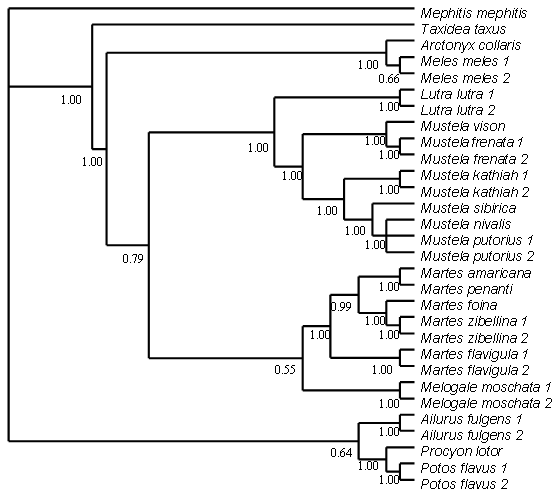


**Plod2-14**

**Ssr1-5**


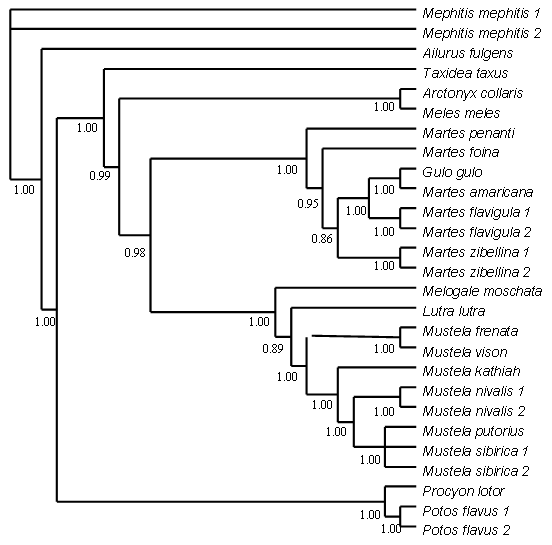

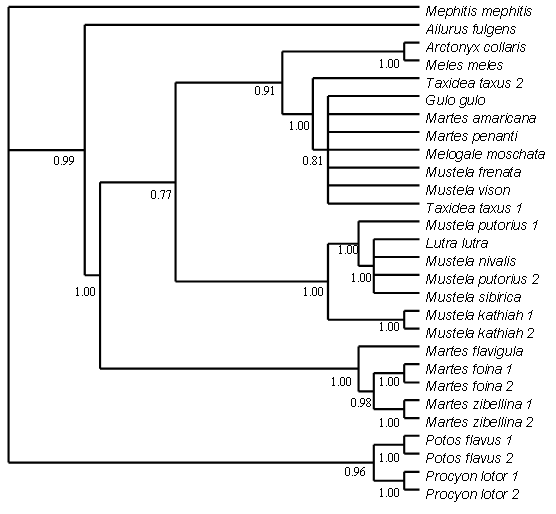


**Tbc1d7-6**

**Tinagl1-1**


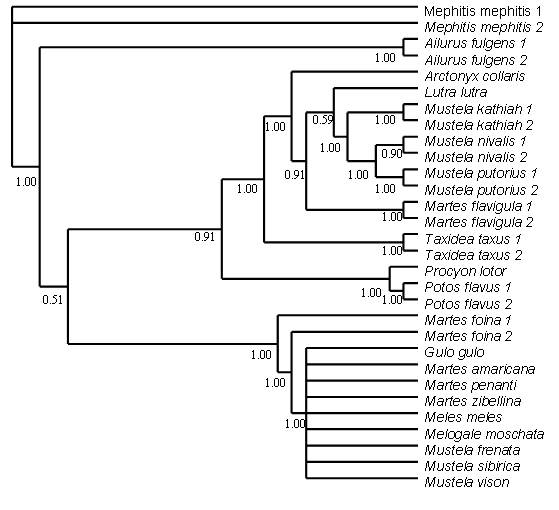

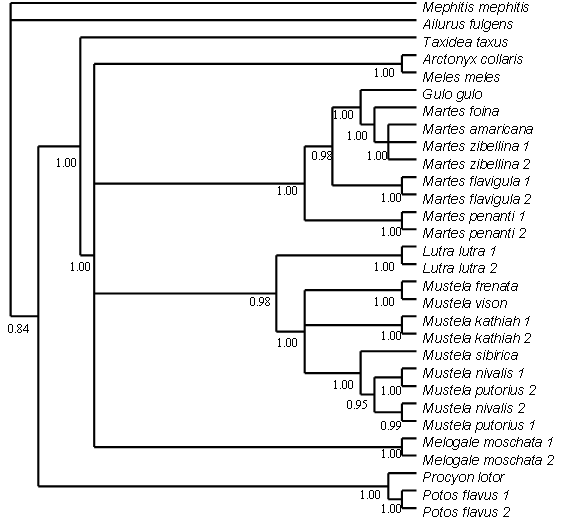


**Tinagl1-3**

**Ttr1**


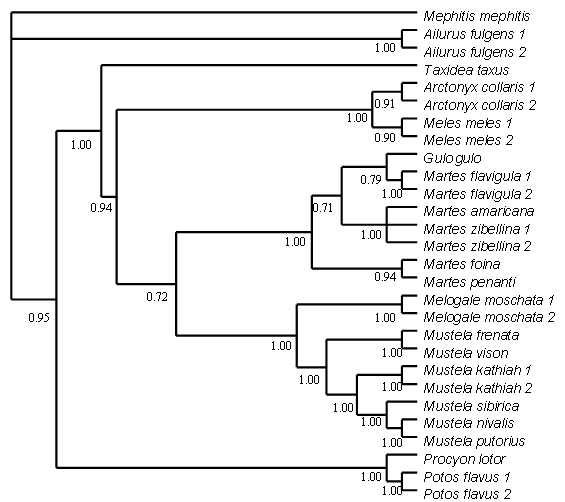

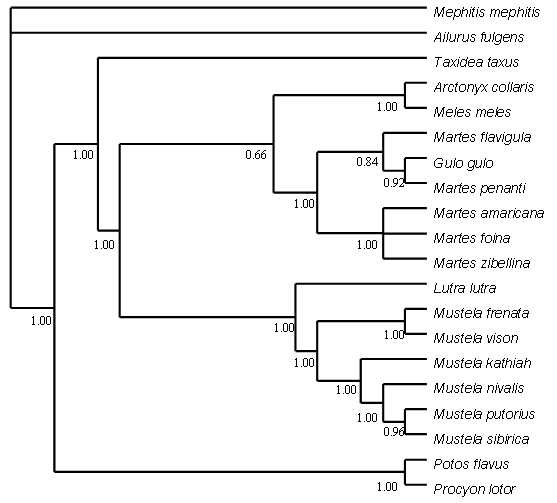


**Wasf1-3**

**Wasf1-6**


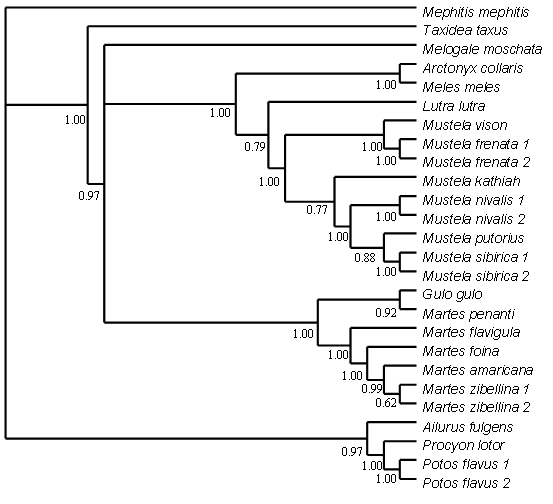

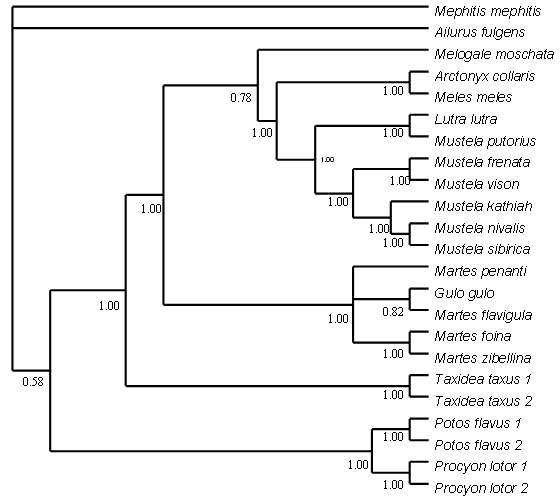


**Wasf1-7**


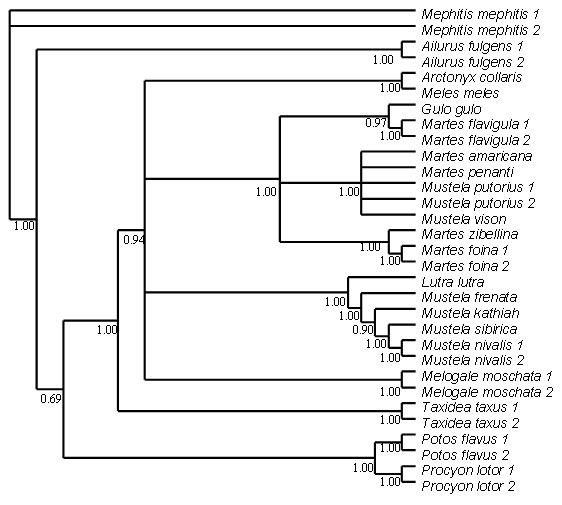

Supplement: Additional file 1 — Phylogenetic relationships of Mustelidae based on the analyses of 17 single introns. The IIAHs within a species was shown as 1 and 2. All trees shown were reconstructed using Bayesian method. Posterior probabilities (PP) are shown above internal nodes. [file 1471-2148-11-92-S1.DOC]
