# Supplementary figures and images for: On the phylogeny of Mustelidae subfamilies: analysis of seventeen nuclear non-coding loci and mitochondrial complete genomes
Source: BMC Evol Biol. 2011 Apr 10;11:92. doi: 10.1186/1471-2148-11-92 (PMC3088541; doi:10.1186/1471-2148-11-92)

**Additional file 2**


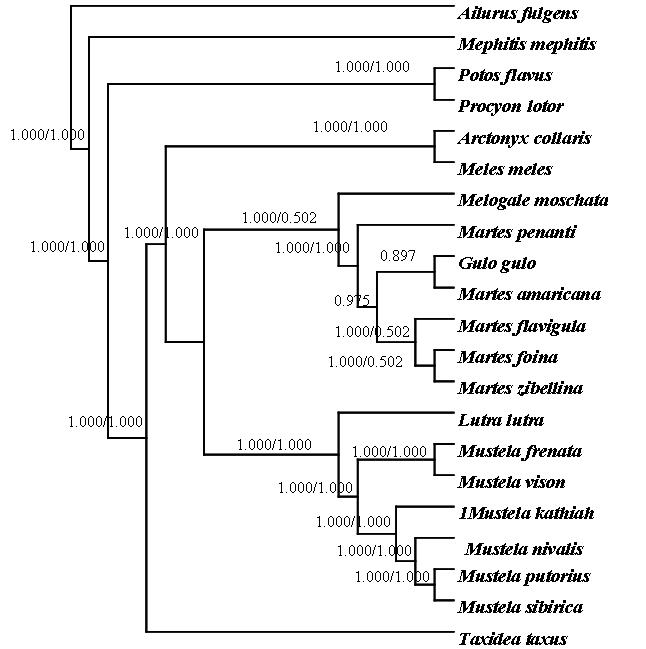

Supplement: Additional file 2 — Phylogenetic relationships of Mustelidae based on the Bayesian concordance analysis (BCA) of the nuclear intron gene datasets and the nuclear plus the mt genome datasets. The concordance factors (CFs) from the nuclear intron gene analysis and the nuclear plus the mt genome analysis are shown above internal nodes. [file 1471-2148-11-92-S2.DOC]
