# Supplementary material for: On the phylogeny of Mustelidae subfamilies: analysis of seventeen nuclear non-coding loci and mitochondrial complete genomes
Source: BMC Evol Biol. 2011 Apr 10;11:92. doi: 10.1186/1471-2148-11-92 (PMC3088541; doi:10.1186/1471-2148-11-92)

**Additional file 3**

Phylogenetic relationships of Mustelidae based on the analyses of 13 individual protein-coding genes, 2 individual tRNA genes, 22 tRNAs, combined protein-coding genes, and combined tRNA genes. All trees shown were reconstructed using Bayesian method. Posterior probabilities (PP) are shown below internal nodes.

**13 Coding genes ND1**


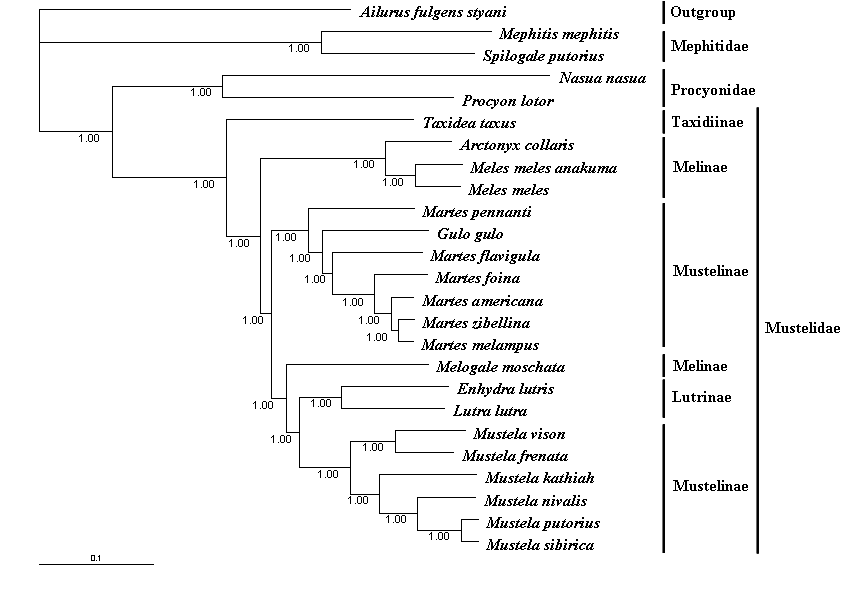

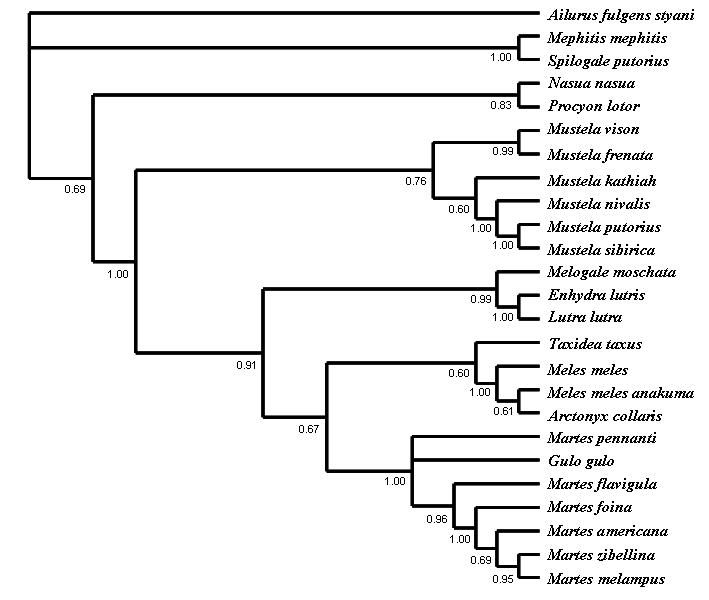


ND2 COX1


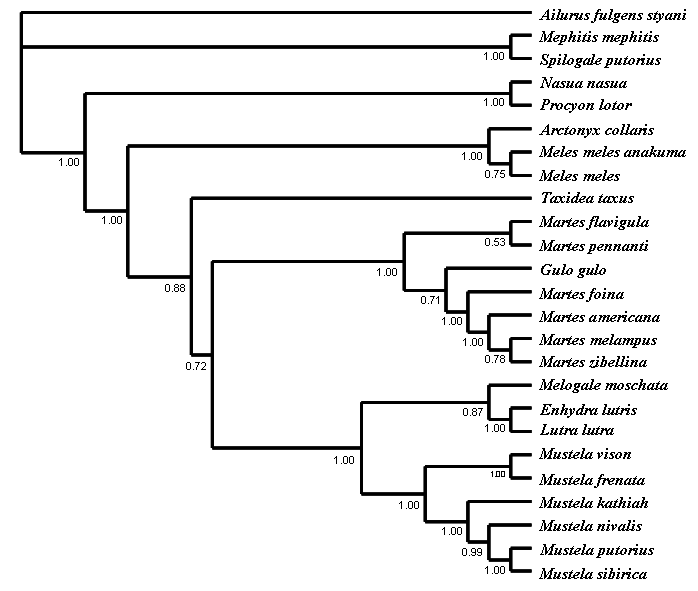

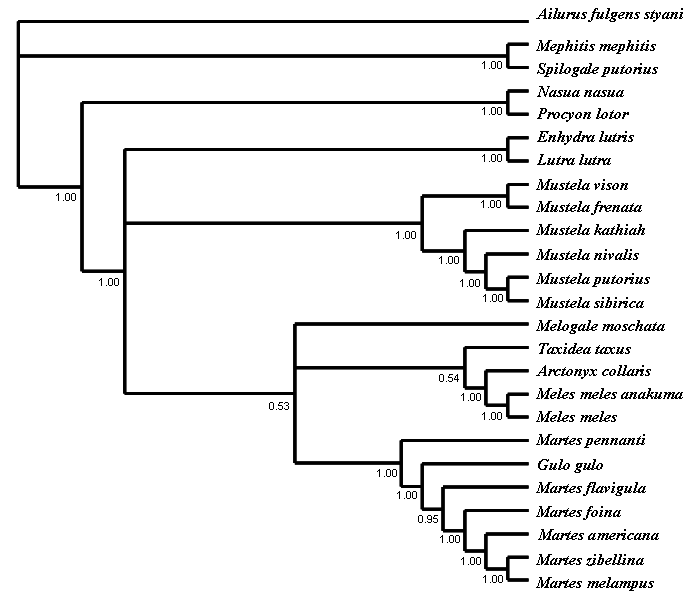


COX2 ATP8


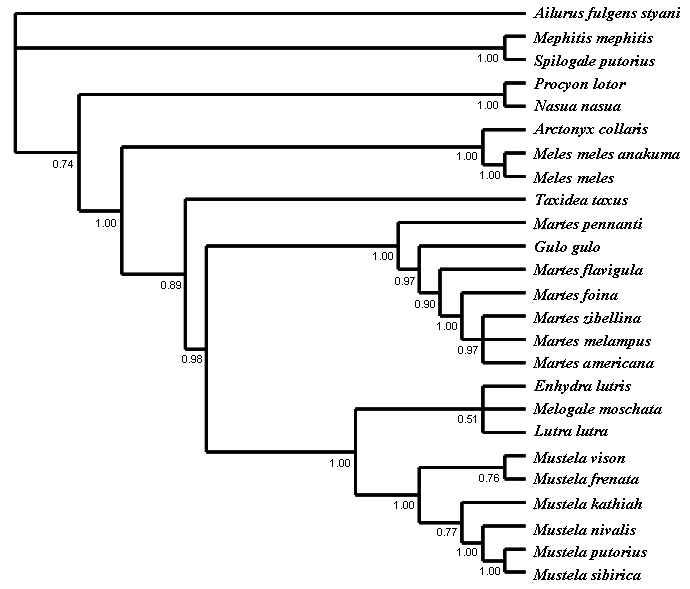

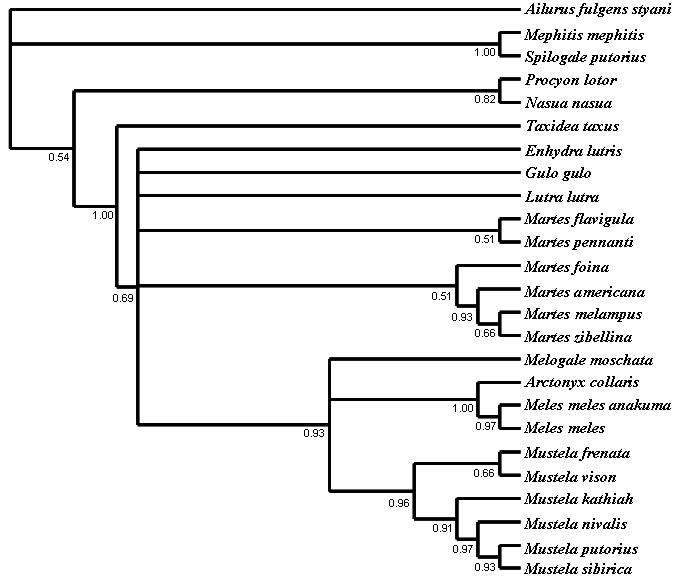


ATP6 COX3


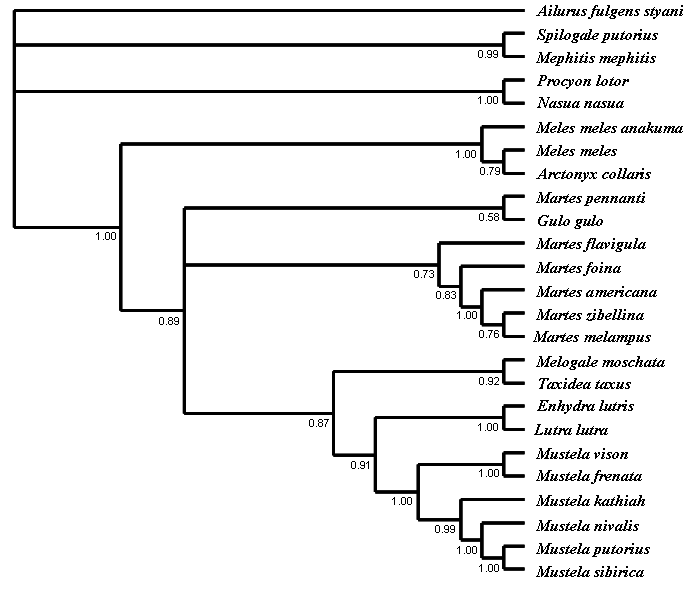

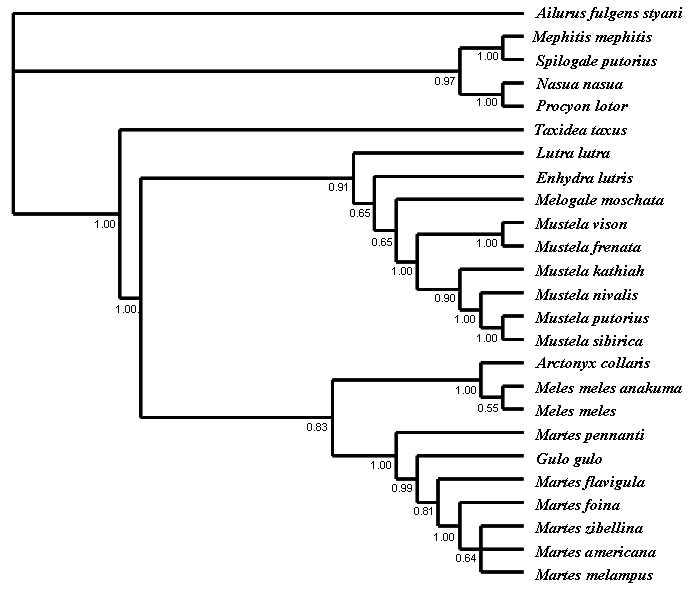


ND3 ND4L


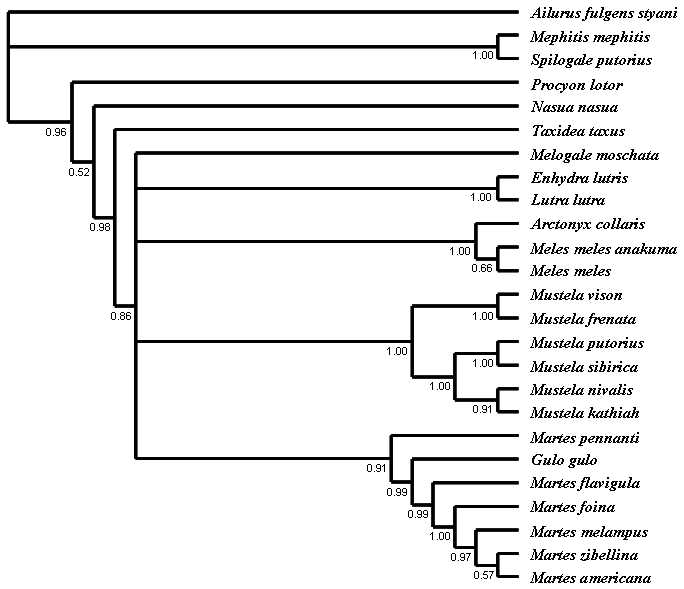

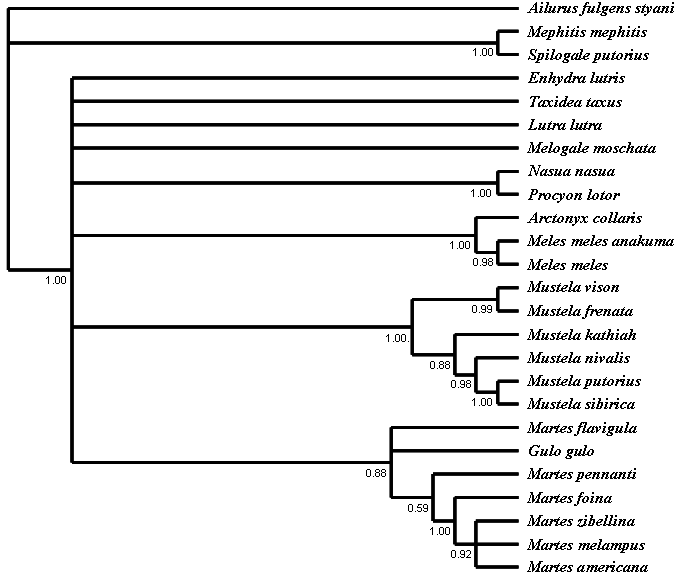


ND4 ND5


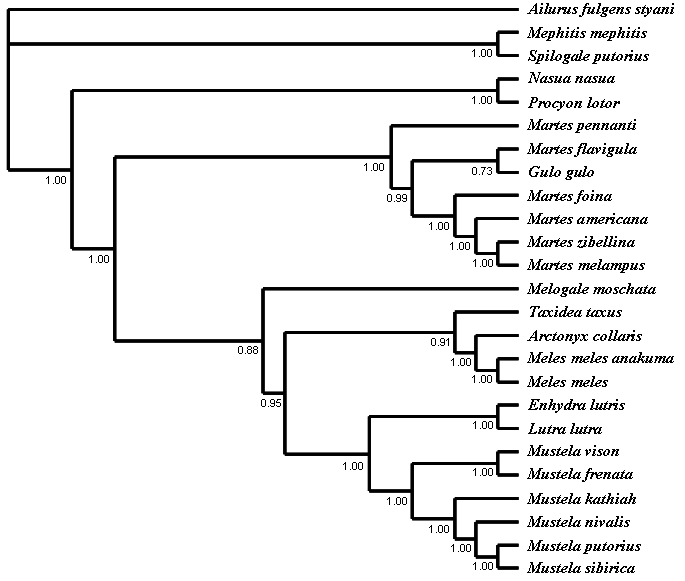

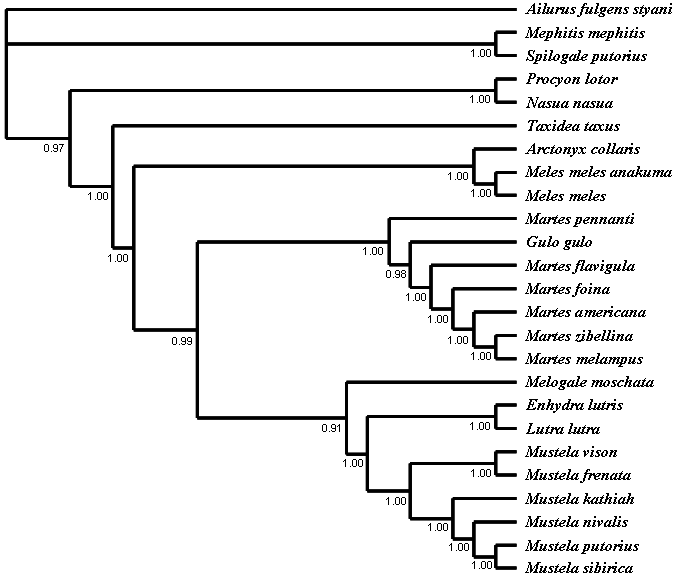


ND6 CYTB


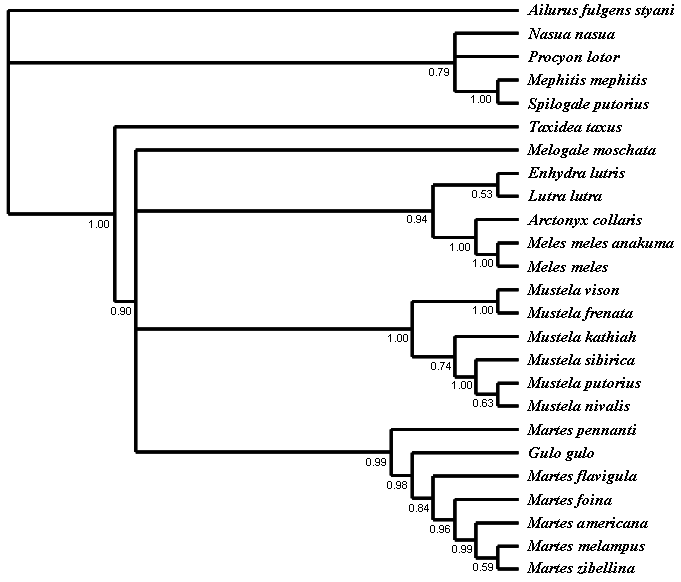

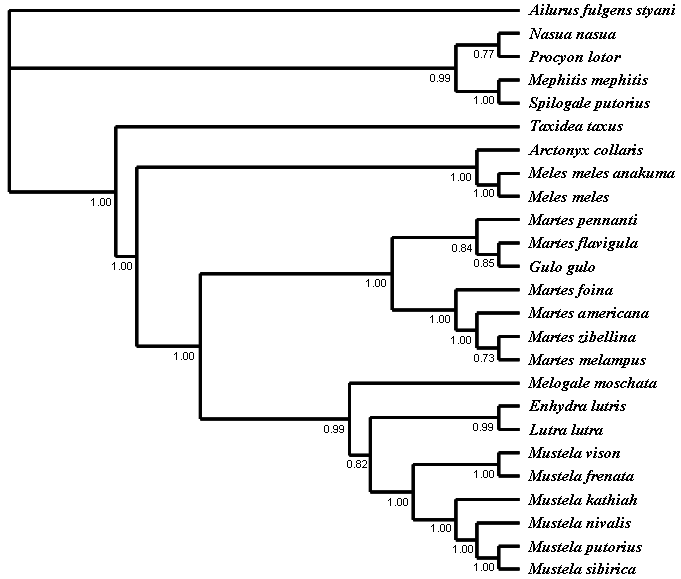


12S 16S


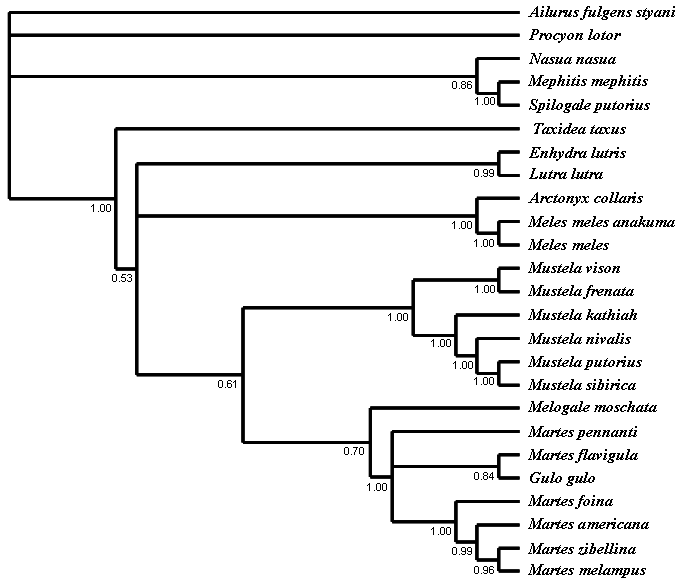

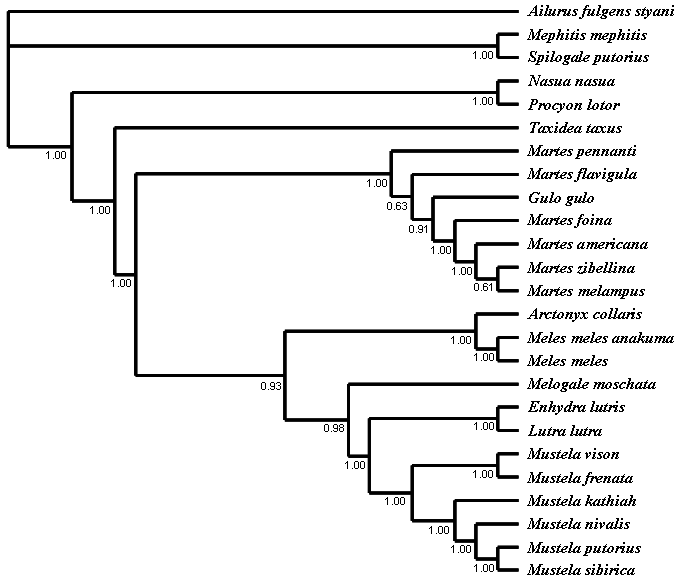


12S-16S tRNA


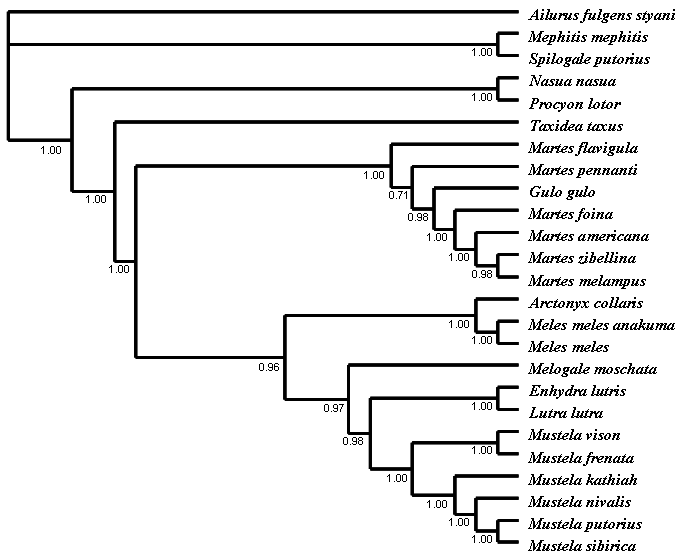

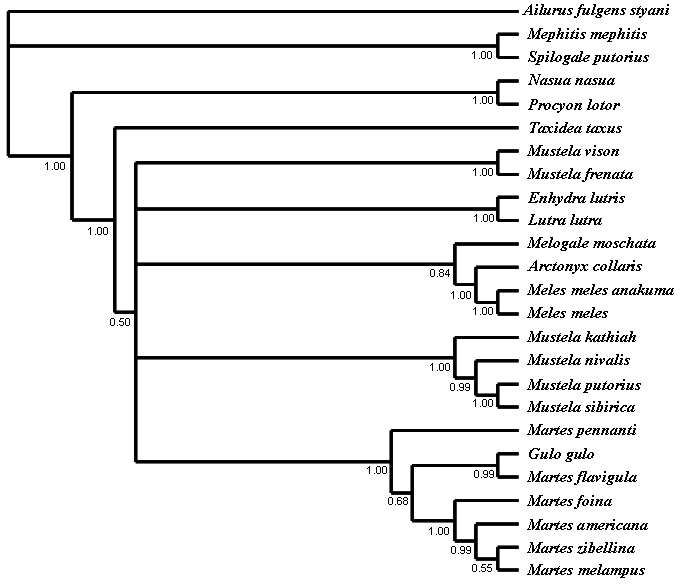

Supplement: Additional file 3 — Phylogenetic relationships of Mustelidae based on the analyses of 13 individual protein-coding genes, 2 individual rRNA genes, 22 tRNAs, combined protein-coding genes, combined rRNA genes, and combined tRNA genes. All trees shown were reconstructed using Bayesian method. Posterior probabilities (PP) are shown above internal nodes. [file 1471-2148-11-92-S3.DOC]
